# Unusual presentation of miliary tuberculosis in a 12-year-old girl: a case report

**DOI:** 10.1186/s12887-023-04427-x

**Published:** 2024-04-01

**Authors:** Mahsa Kamali, Mohammad Reza Navaeifar, Ali Abbaskhanian, Azin Hajialibeig, Farnaz Godazandeh, Mahsa Salehpour, Mohammad Sadegh Rezai

**Affiliations:** 1 Pediatric Infectious Diseases Research Center, Communicable Diseases Institute, Mazandaran University of Medical Sciences, Sari, Iran; 2 Department of Radiology, Faculty of Medicine, Mazandaran University of medical sciences, Sari, Iran

**Keywords:** Miliary, Tuberculosis, Mycobacteria

## Abstract

**Background:**

Miliary tuberculosis (TB) is a lethal hematogenous spread form of mycobacterium tuberculosis with approximately 15–20% mortality rate in children. The present report highlights the clinical manifestations of an unusual presentation of miliary tuberculosis in a 12-year-old girl.

**Case presentation:**

In this case, extensive lung involvement was presented despite the absence of respiratory symptoms. Also, some central hypo-intense with hyper-intense rim nodules were detected in the brain’s pons, right cerebral peduncles and lentiform nucleus.

**Conclusion:**

The results of this study showed that severe miliary TB may occur even in a person who received the Bacille Calmette-Guérin (BCG) vaccine.

## Background

Tuberculosis (TB), caused by mycobacteria, is a preventable and curable disease with 1.5 million mortality annually most of them live in low and middle-income countries [1]. Intracranial tuberculosis (ITB) is an unusual and rare presentation of extrapulmonary tuberculosis [2]. The incidence of ITB is 5–30% of all intracranial lesions [3]. Nevertheless, it often remains underestimated [4]. Disseminated TB and miliary TB had similar pathogenesis but the anatomical-pathological findings are different [5]. Disseminated TB is an important cause of mortality and morbidity in children under 15 years old especially in developing countries [6]. Disseminated TB describing as entering the bacteria into the systemic circulation, then they multiply and infect extrapulmonary organs [7]. They account for highly variable clinical manifestations including fever, weight loss, anorexia and nocturnal sweating [8]. Miliary TB is a lethal hematogenous spread form of mycobacterium tuberculosis to several organs, diagnosed by the presence of a diffuse miliary infiltrate on a chest X-ray, CT scan and pathological evidence [9]. Peripheral lymphadenopathy and hepatosplenomegaly are the most common childhood military TB signs. The mortality rate of childhood miliary TB is approximately 15–20% [10, 11]. As a huge challenge, the nonspecific clinical features of miliary TB often result in delayed diagnosis followed by a poor prognosis condition [12]. Also, this clinically silent TB leads to inadequate treatment in young children [13]. So, in children, considering ITB as a differential diagnosis is important. The present case report highlights the clinical presentation of unusual miliary TB evidence in a 12 years old girl.

## Case presentation

A 12-year-old girl was admitted to a general hospital, in one of the western cities of Mazandaran province, with chief complaints of muscle atonia, and foam coming from the mouth for 20 s following fever and transient left hemiparesis. Ten minutes later, she agitated and presented generalized tonic-clonic movements lasting for 15 min and decreased consciousness following urinary incontinence. During this episode, she didn’t have foam coming from her mouth. She was intubated immediately and transferred to the PICU (Pediatric intensive care unit) of a tertiary hospital, in Sari, Mazandaran province. She had been visited by a general physician due to fever (T = 38.5 ^o^c), vomiting (digested food particles and non-bloody), diplopia and headache one week before hospitalization and received symptomatic treatment. With relative recovery, the signs and symptoms aggravated the night before hospital admission. Her family reported unintentional weight loss accompanying anorexia and weakness from two months ago, but additional imaging was not performed.

Medical history and physical examination were as follows: The initial vital signs were blood pressure: 105/70 mmHg, the pulse rate: 110 beats per minute, respiratory rate: 22 per minute, axillary temperature: 37.4 ^o^c and SPO2: 98% (intubated). Skin: Negative in terms of petechia, purpura and ecchymosis. Eyes: pale conjunctiva. Extremities: 1 + deep tendon reflexes. The tone and power of muscle were normal and there were not any signs of cerebellar or basal ganglion involvement. Lymph node: No lymphadenopathy. Chest and lung: No chest deformity, she was intubated, and symmetric lung sounds. Abdomen: No distention and organomegaly. No sign and symptom of increased intracranial pressure. She had no history of contact with Coronavirus disease 2019 (COVID-19) patients, contaminated water, rice field and trauma. She had fainted in her childhood period following excessive activity (medical follow-up showed cardiac chamber defect) and improved spontaneously when she was 3 years old. She completed all doses of recommended childhood vaccines but did not receive the COVID-19 vaccine. She had a drug allergy to Cefixime and penicillin and a food allergy to eggplant. Initially, her family did not report a positive family history of TB, but after the final diagnosis, they declared that her uncle died following TB 3 years ago.

**Fig. 1:**
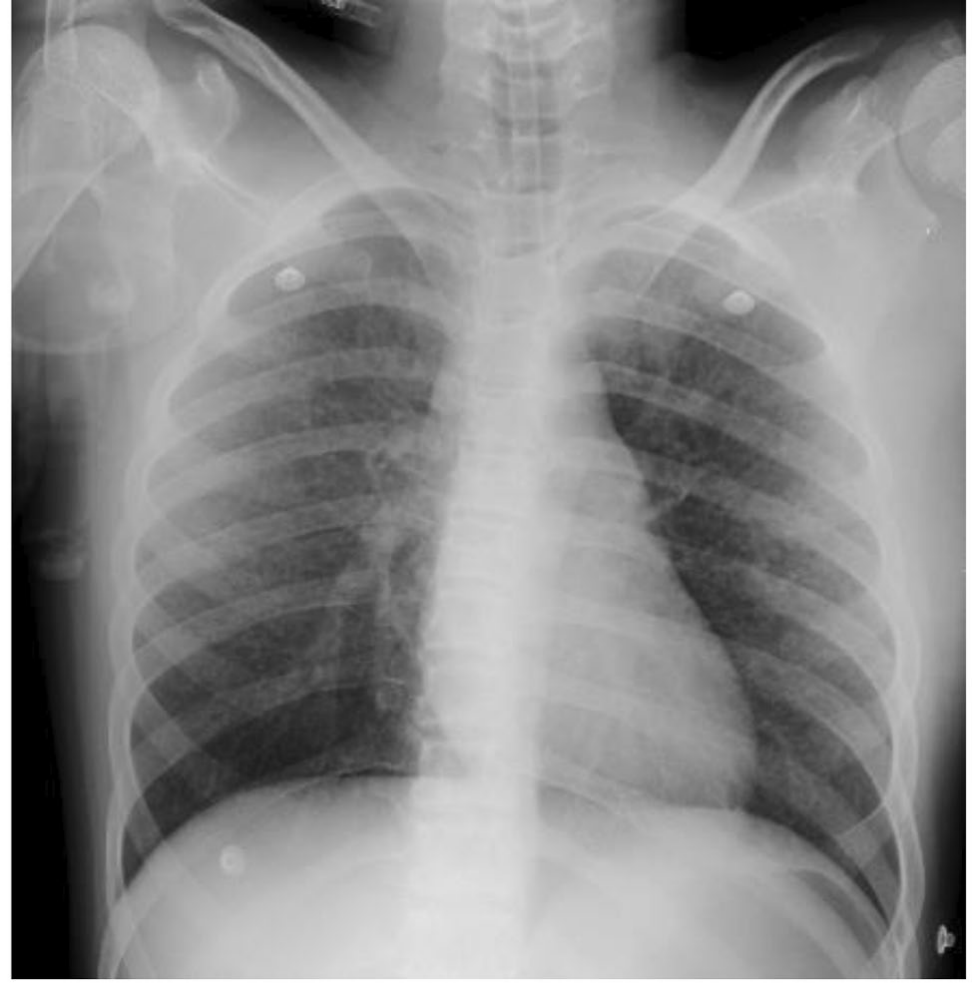
CXR. PICU admission: AP chest x-ray demonstrates wide spread tiny nodular opacities distributed throughout both lungs

**Fig. 2:**
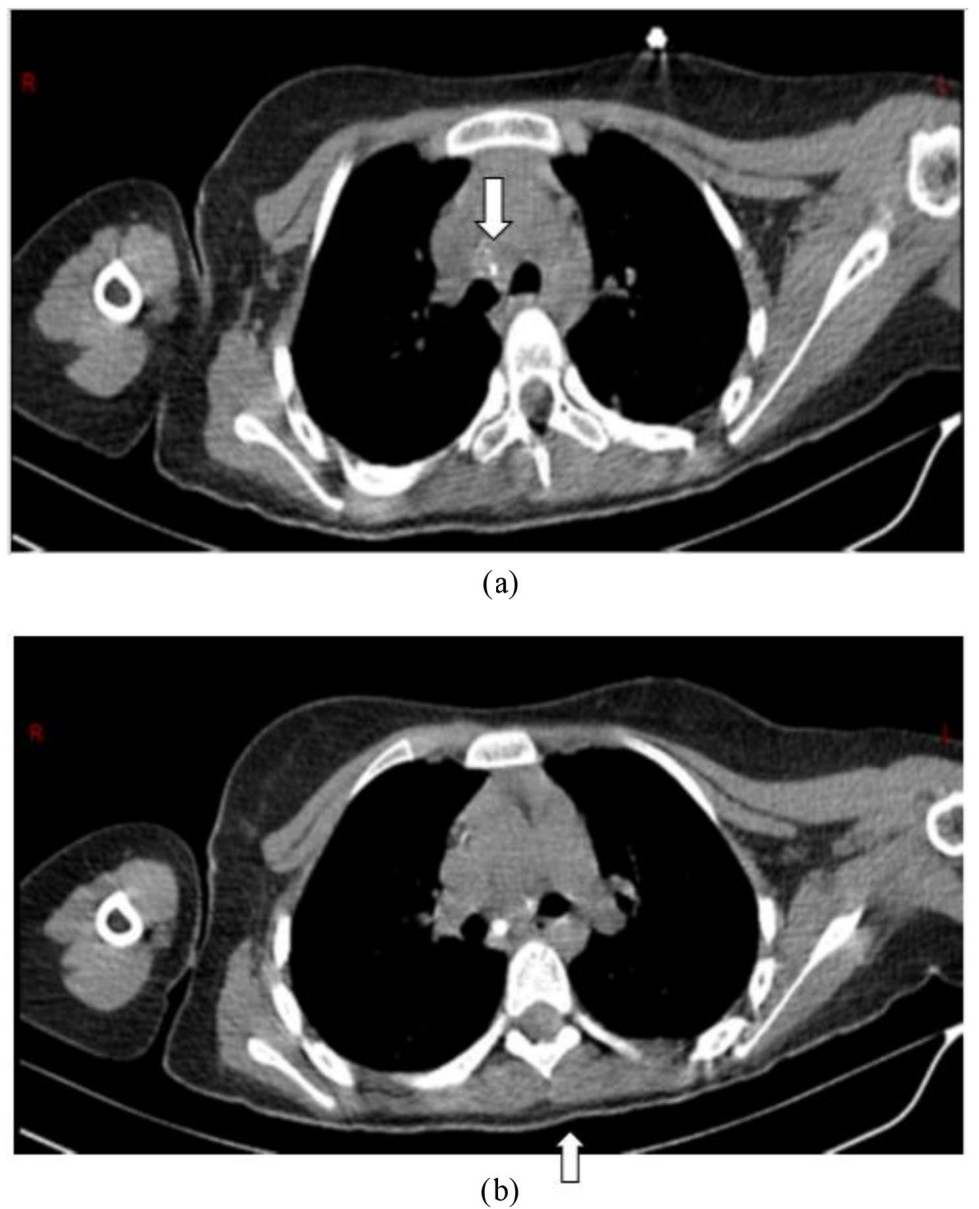
**a, b**) Chest CT scan without contrast (mediastinal window): Some calcified lymphadenopathies in the right paratracheal and subcarinal space of the middle mediastinum (white arrows)

**Fig. 3:**
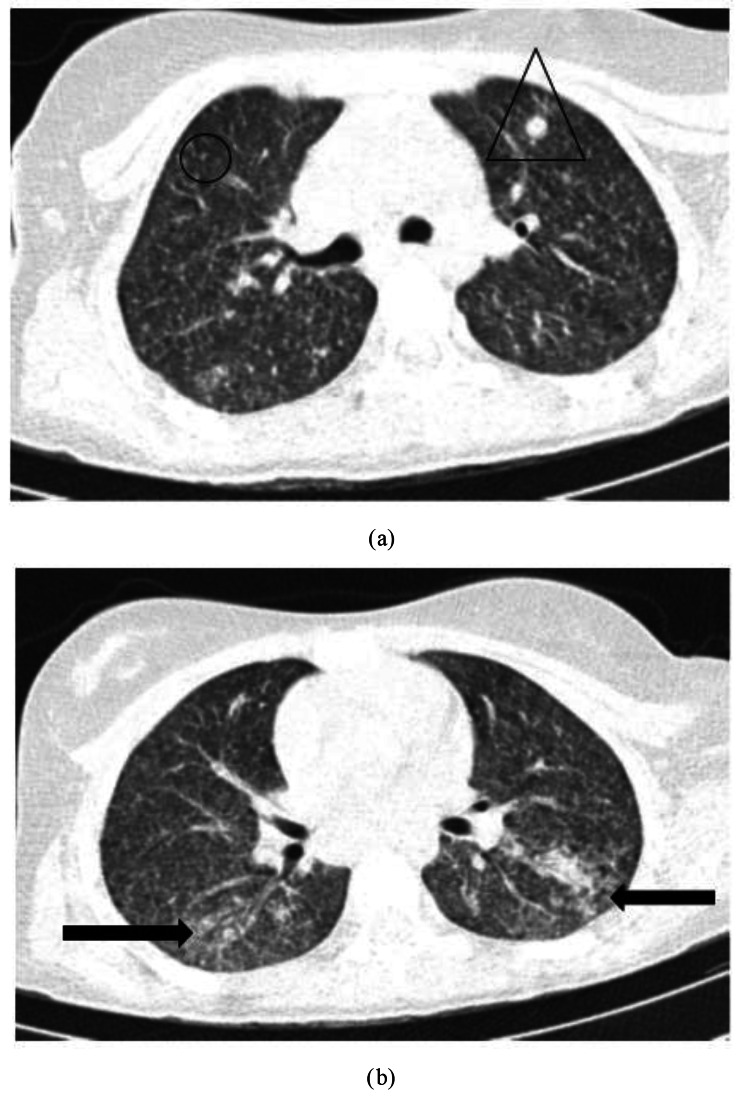
**a, b**) Chest CT scan without contrast (Lung window): Diffuse multiple miliary nodules (black circle) and some scattered micronodules in a random distribution (black triangle) in both lungs associated with patchy consolidation (black arrows) in lower lobes, in favor of pulmonary TB

**Fig. 4:**
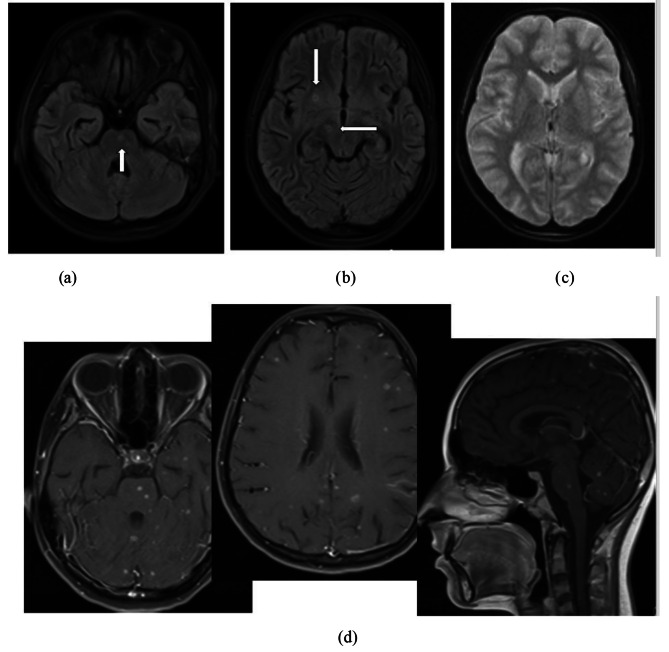
Brain MRI without contrast: 4-**a** & 4-**b**) Flair sequence: some central hypo-intense with hyper-intense rim nodules in the pons, right cerebral peduncles and lentiform nucleus (white arrows). 4-**c**) DWI/ADC: no restricted diffusion. 4-**d**) Contrast–enhanced MRI: Diffuse Multiple rim-enhancing nodules in both cerebral, cerebellar hemispheres and brainstem. These findings are suggestive of caseating tuberculomas with tuberculous meningitis. DWI: Diffusion-weighted imaging – ADC: Apparent diffusion coefficient

Para-clinical investigations: The result of COVID-19 Reverse transcription polymerase chain reaction (RT-PCR) and galactomannan level was negative. Also, the levels of HIV (human immunodeficiency virus) antibody, Venereal disease research laboratory (VDRL), CD4 (cluster of differentiation 4), CD19 and Complement hemolysis (CH50) were normal. The echocardiography showed mild Tricuspid valve regurgitation and mild pulmonary valve insufficiency. Also, there was no pulmonary hypertension. Based on the possible COVID-19 bilateral involvement in chest X-ray and lung CT scan, Pro-BNP, D-dimer and troponin levels were requested. Only the D-dimer level was high. It was 1710. The lumbar puncture (LP) was done on the first day of the PICU admission. The Cerebrospinal fluid (CSF) analysis results are presented in Table 1. Although, the girl had no signs and symptoms of meningitis including Brudzinski’s and Kernig’s signs, there was CSF involvement. CSF analysis showed increased levels of protein and WBC. The patient had not the coughing or sputum, so the gastric aspirate culture PCR (three times) had been done and the result was positive for miliary TB. Findings of the chest X-ray (Fig. 1), lung CT scan (Figs. 2 and 3) and brain MRI (Fig. 4) were suggestive of caseating tuberculomas. Finally, the miliary TB was diagnosed based on brain MRI, positive gastric aspirate culture and also two organs involvement. AP chest x-ray demonstrates wide spread tiny nodular opacities distributed throughout both lungs. The PPD test were normal.

A pediatric infectious diseases specialist prescribed rifampin, ethambutol, pyrazinamide, isoniazid and vitamin B6 (For prevention of the side effect of isoniazid) based on the miliary TB evidence in MRI. The anti-tuberculosis medication dosage was adjusted due to elevated AST and ALT levels 10 days later. So, the rifampin and isoniazid were discontinued and in the follow-up due to the normal levels of AST and ALT, the rifampin and isoniazid were added to the medication regimen. At the time of discharge, approximately one month later, the gastric aspiration result was negative. She was discharged in stable condition with fixed-dose combination anti-TB medication (III), acid folic and vitamin B6 tablets daily. On her follow-up, AST and ALT levels were normal. Also, her medication changed to two-drug formulations. The girl had been follow-up for at least one year and the MRI finding and gastric aspirate on follow-up were normal.

**Table 1:** The results of the CSF analysis

CSF finding		Baseline	Two days later
CSF smear	Appearance	Clear	Semi-clear
	Color	Colorless	Light red
	WBC	12	50
	RBC	15	5000
	Glucose	39	21
	Protein	272	370
CSF culture		Negative	Negative
CSF PCR for herpes		Negative	Negative

## Discussion

In the present case report, the miliary TB evidence was presented in a 12 years old girl. Miliary TB has been more prevalent in middle age and older people than children [14, 15]. Also, 60% of the cases are male with a mean age of 55 years in studies [16–18]. Although the mean age of miliary TB patients has increased, the rates remain relatively low in children [19, 20]. In the present study, the unusual miliary TB pattern was seen in a 12 years old girl. The miliary TB in young patients is reported in TB-endemic countries [21]. Based on the results of a retrospective descriptive study in South Africa, 32.7% of children were TB meningitis. Also, the recent study reported 43.3% of children drug resistant was the main reason for hospitalization [22]. But, in our case, the girl had no drug resistance. Approximately 13% of the TB cases suffer from HIV infections [23]. TB becomes more generalized and affects more than one organ when progressive immunosuppression occurs [24]. However, the present case didn’t have HIV infection as co-infection or other immunodeficiency condition. Corticosteroid therapy can reactivate cryptic TB [25]. Song et al. reported a 36-year-old man who underwent corticosteroid therapy and two weeks later, he experienced an acute exacerbation of miliary TB [26]. But in our study, the patient had no history of recent corticosteroid therapy. Considering increased the number of BCG-vaccinated children and improving the diet status of children, we see the modified clinical profile of neurotuberculosis including TB meningitis nowadays with wide varieties of clinical manifestations according to the site of the brain lesion [27]. The clinical presentation of neurotuberculosis may be nonspecific leading to delayed treatment and poor clinical outcome [28]. The present case had diffuse multiple rim-enhancing nodules in both cerebral, cerebellar hemispheres and brainstem in the brain. The brain and central nervous systems are two target organs of mycobacterium tuberculosis that cause serious and dangerous forms of extrapulmonary tuberculosis [29]. The most obvious initial clinical symptoms of our case were fever, weight loss, anorexia and seizure. Other studies reported fever, cough, seizure, diarrhea, hepatomegaly, splenomegaly, jaundice, anorexia and weight loss as the most common clinical presentations in children [6, 30, 31]. A male three-months Brazilian infant was admitted with nocturnal fever, sweating and coughing 10 days before hospitalization and he was not responsive to antibiotic therapy. The results of the chest X-ray showed bilateral miliary TB and also CNS TB based on the CT-scan report [32]. Similar to our case, the recent case showed early diagnosis and treatment in endemic areas. Delayed diagnosis in miliary TB patients can cause serious complications including cranial nerve involvement, convulsions and death [16] as a seizure occurred in our case. Machida et al.’s report showed 1% of TB patients had CNS involvement which is about a high mortality rate and permanent neurological sequelae [33]. Fortunately, in the present case, follow-up interventions showed no neurological sequelae and she was in good general condition. Multiple diagnostic tests are provided to detect miliary TB, including PCR, sputum smear and acid-fast staining, in addition to histopathological findings but radiology plays a major role in this regard [34]. In this case, despite the absence of respiratory symptoms and non-significant lung involvement in chest X-ray, extensive lung involvement was seen including diffuse multiple miliary nodules. Also, a chest CT scan revealed some scattered micronodules in a random distribution in both lungs associated with patchy consolidation in lower lobes in favor of pulmonary TB. Additionally, in the brain, some central hypo-intense with hyper-intense rim nodules in the pons, right cerebral peduncles and lentiform nucleus, multiple diffuse rims enhancing nodules in both cerebral, cerebellar hemispheres and brainstem were obvious.

The COVID-19 pandemic has emerged new conditions, including neuro-COVID which has presented by lung involvement and seizure [35]. During the COVID-19 pandemic, miliary TB should be considered in TB endemic areas due to neuro-COVID diagnosis.

Our study showed that teenage patients might present extensive lung involvement in favor of miliary TB even in the absence of respiratory symptoms. Also, we found that severe miliary TB may occur even in a person who received the bacille Calmette-Guérin (BCG) vaccine. In an Iranian study, in 15 children aged under 72 months, disseminated BCG infection occurred after BCG vaccination [36]. Also, in patients with CNS symptoms including seizure especially in TB endemic areas, miliary TB should be considered as a differential diagnosis, to prevent delay in diagnosis and treatment. We live in the TB endemic area. So, when a patient is admitted with a decreased level of consciousness and there is no reasonable cause, we should consider TB as a differential diagnosis. In the current case report, due to early diagnosis and treatment, neurological sequelae were not observed.

## Data Availability

Due to the privacy of the patients, the data generated during the current study are not publicly available but are available from the corresponding author upon reasonable request.
